# Hypertrophic Cardiomyopathy with Biventricular Involvement and Coronary Anomaly: A Case Report

**DOI:** 10.3390/life12101608

**Published:** 2022-10-14

**Authors:** Ylenia Bartolacelli, Simone Bonetti, Anna Balducci, Ambra Bulgarelli, Luca Ragni, Andrea Donti

**Affiliations:** Pediatric Cardiology and Adult Congenital Heart Disease Program, Department of Cardio-Thoracic and Vascular Medicine IRCCS Azienda Ospedaliero-Universitaria di Bologna, 40122 Bologna, Italy

**Keywords:** hypertrophic cardiomyopathy, right ventricular obstruction, cardiac imaging

## Abstract

Although hypertrophic cardiomyopathy (HCM) is classically considered a disease of the left ventricle, right ventricular (RV) involvement has also been reported, though still not extensively characterized. We present a case of biventricular HCM with significant RV involvement in the absence of a left intraventricular gradient: RV outflow tract gradient due to hypertrophy and near obliteration of the RV cavity. Significant RV hypertrophy may cause reduced RV diastolic filling and/or RV outflow obstruction, with potentially increased incidence of symptoms of heart failure, arrhythmias, and pulmonary thromboembolism. The optimal treatment for these patients is unclear. Our patient underwent complete treatment and elimination of right ventricular obstruction, resulting in improved symptoms and a significant reduction in postoperative gradients. Direct relief of outflow tract obstruction can be achieved with low morbidity and good intermediate- to long-term results. Conventional surgery may provide significant symptomatic improvement and should thus be considered in the setting of HCM with outflow obstruction.

## 1. Introduction

The 2020 American Heart Association and American College of Cardiology guidelines for the diagnosis and treatment of patients with hypertrophic cardiomyopathy (HCM) define HCM as “a disease state in which morphologic expression is confined solely to the heart, predominantly by left ventricular hypertrophy (LVH) in the absence of other systemic or metabolic disease capable of producing the magnitude of hypertrophy evident in a given patient and for which a disease-causing sarcomere (or sarcomere-related) variant is identified, or genetic etiology remains unresolved” [1]. In particular, clinical diagnosis is established by imaging, evaluating maximal wall thickness in the left ventricle. Right ventricular (RV) hypertrophy is usually considered as part of other morphological abnormalities not diagnostic of HCM but considered part of phenotypic expression of the disease [1].

Therefore, RV involvement is still not extensively characterized and has been explored to a lesser degree. We present a case of biventricular HCM with significant RV involvement.

## 2. Case Description

A 6-month-old child came for cardiological evaluation due to the finding of a heart murmur during a routine pediatric evaluation. His father had a known hypertrophic cardiomyopathy and had an ICD for primary prevention. He had undergone genetic evaluation reporting sarcomeric causative mutation but, unfortunately, he never shared the complete results and was followed-up by another institution. The patient’s grandfather from the paternal side died at a young age and had the same mutation. No other relative in the family was affected by the disease. The child underwent a complete echocardiogram that showed a hypertrophic cardiomyopathy with moderate right ventricular obstruction; thus, he was started on propranolol and a regular cardiological follow-up was planned every six months. The child underwent genetic evaluation that excluded metabolic abnormalities.

Due to progressive increased right ventricular outflow obstruction with maximum gradient 130 mmHg, he underwent surgery (right medio-ventricular resection) at the age of 5 and continued therapy with oral beta-blockers.

**Figure 1:**
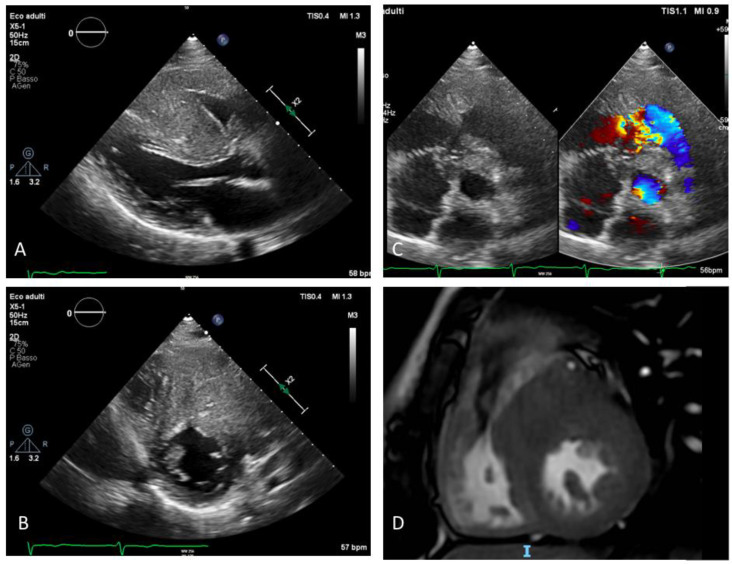
Preoperative imaging showing severe biventricular hypertrophy with right ventricular outflow tract obstruction: (**A**) Parasternal long axis view: severe hypertrophy of interventricular septum and of the RV free wall; (**B**) parasternal short axis view: severe hypertrophy of RV outflow tract and color Doppler aliasing due to restriction of outflow tract and consequent flow acceleration (**C**) parasternal short axis view: severe and asymmetric hypertrophy of IVS and RV free wall, (**D**) CMR focused on RV outflow tract: hypertrophy of the RV free wall and IVS. RV: right ventricle; IVS: interventricular septum; CMR: cardiac magnetic resonance.

During the last routine clinical assessment, at the age of 15, the patient underwent a cardiac MR (Figure 1) which showed severe left ventricular hypertrophy (maximal thickness of 32 mm in the anterior septum), right free wall ventricular hypertrophy (maximal thickness of 11 mm in the infundibulum) with right outflow obstruction (infundibular stenosis) and focal DE of the medium septum.

An echocardiogram (Figure 1) showed a marked right ventricular hypertrophy with trabecular salience and apical obliteration, right ventricle dilatation with reduced systolic function (FAC 32%, TAPSE 15 mm, RV dP/dt 568 mmHg/s, TDI s’ 11 cm/sec) and medio-ventricular obstruction (maximum gradient 120 mmHg, relatively symmetric dome-like curve) caused by a muscular spur at the infundibular level. The left ventricle was severely hypertrophic (eccentric hypertrophy with maximal thickness of 36 mm) with normal global systolic function and without left outflow obstruction (both in basal conditions and after Valsalva maneuver). Diastolic mitral pattern was restrictive (E/E’ 15, deceleration time 137 ms) and mitral regurgitation was absent.

When clinically assessed, the patient complained of progressively worsening dyspnea on exertion during the previous months but no history of tachycardia or syncope. On physical examination, he had a regular pulse (55 bpm) with a blood pressure of 120/70 mmHg. The oxygen saturation was 99% in room air. He had elevated jugular venous pressure and a systolic heart murmur (4/6 L). His chest was clear on auscultation. There was no peripheral oedema present. A 12-lead electrocardiogram showed sinus rhythm at a rate of 55 bpm, with a marked left axis deviation, signs of biventricular hypertrophy and right ventricular bundle branch block.

Due to these findings, the patient underwent implantation of an epicardial ICD for primary prevention and, after few months, this was followed by right ventricular muscular resection.

The postoperative course after the resection was without complications. After surgery, the patient was found to be hypertensive and an ACE inhibitor was started with clinical benefit. Before drainage removal, due to a massive serum loss, a therapy with corticosteroids was started. Therapy at discharge after 10 days was metoprolol 100 mg twice daily, ramipril 10 mg once daily and prednisone 25 mg twice daily.

A two-week follow up was unremarkable and the patient was asymptomatic. His physical examination revealed normal blood pressure (120/70 mmHg), 100% oxygen saturation in room air, normal venous pressure, mild systolic–diastolic heart murmur (2/6L) and a clean surgical wound. A chest radiography was unremarkable.

**Figure 2:**
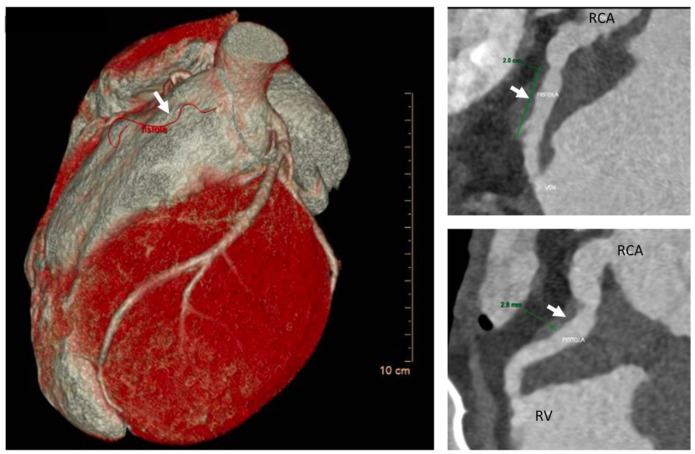
AngioTC and volume rendering image: Infundibular branch of large caliber originating from CDX; after an intramyocardial course involving the proximal tract (length 2 cm and depth 3 mm) to the middle tract, it fistulizes with the right ventricular outflow tract. White arrow is pointing at the fistula RCA = right coronary artery.

An echocardiogram demonstrated marked left ventricular hypertrophy with mild hypokinesia of the left ventricular septum, mildly reduced global ejection fraction (48%), mild eccentric mitral regurgitation and absence of left ventricular obstruction. The right ventricle was hypertrophic with mildly reduced systolic function, no right ventricular obstruction, and mild tricuspid regurgitation with an estimated right ventricular pressure of 30 mmHg. A systolic–diastolic flow was noted in the right ventricle, at the level of the medium right ventricular coronary artery tract, indicating a possible fistula between the right coronary artery and the right ventricle (maximum gradient 80 mmHg). A heart computed tomography (CT) scan was performed, showing an intramyocardial course of the proximal anterior intraventricular artery and a fistula (Figure 2) between the infundibular coronary branch arising from the right coronary artery and directed to the right ventricular outflow tract.

After multidisciplinary discussion, we decided to continue with clinical follow-up. The patient is currently well, denying chest pain, dyspnea or tachyarrhythmias, NYHA I. No residual outflow obstruction developed. He is continuing with six-monthly outpatient clinical assessment and with therapy with ramipril and metoprolol.

## 3. Discussion

Although hypertrophic cardiomyopathy usually pertains to the left ventricle [2], an involvement of the right ventricle can determine considerable structural and functional changes [3] with significant impact on clinical presentation and prognosis [4].

Based on publications, RV involvement occurs in 28–44% of patients with HCM, depending on the used methods and the relative cut-off values [5]. Histological findings suggest similar pathologic changes in both ventricles, supporting a common myopathic process with sarcomeric mutations leading to myocyte hypertrophy, disarray and fibrosis. The changes are similar but less pronounced in RV, when compared to LV.

The pattern of RV hypertrophy is variable and may include a dynamic obstruction [6] due to the presence of a hypertrophic septum bulging in the RVOT. Moreover, hypertrophy of the infundibulum and RV free wall may contribute to RVOT obstruction. RVOT obstruction has been defined as a pressure gradient exceeding 25 mmHg under resting conditions [7].

Maron et al. suggested that right ventricular outflow tract (RVOT) obstruction is not caused by a dynamic lesion as for classical left ventricular outflow tract (LVOT) obstruction associated with valve systolic anterior motion (SAM). Its principal mechanism is due to a fixed impediment to RV outflow and ventricular cavity reduction due to hypertrophy of the interventricular septum, RV free wall and/or crista supraventricularis. Other authors [8]. Nevertheless, a dynamic obstruction due to tricuspid valve SAM has also been anecdotally described.

Our patient presented with a marked right ventricular free wall hypertrophy with trabecular salience and apical obliteration hypertrophy. The RV obstruction signal appeared relatively symmetric and dome-like, differently from what is usually seen in LVOT obstruction (dagger-shaped), suggesting that the narrowing of the RV cavity by the hypertrophied RV and interventricular septum may contribute to RV obstruction.

According to the literature, RVOT obstruction can occur alone or in combination with LVOT obstruction [5].

The presence of combined RV and LV obstruction can lead to severe diastolic dysfunction with restrictive physiology, and it is worth noting that, sometimes, biventricular involvement is associated with syndromic conditions, such as Noonan syndrome or Pompe disease [4,9,10,11].

Primary affection of RV cardiomyocytes and ultrastructure may lead to RV dysfunction. Other mechanisms affecting RV function are thought to be hemodynamic interaction through backward LV failure or ventricular interdependence. In most cases of HCM with RV dysfunction, a mean pulmonary artery pressure > 35 mmHg was present [12] and post capillary pulmonary hypertension in HCM may depend on increased left atrial pressure due to mitral regurgitation and/or diastolic dysfunction. Ventricular interdependence indicates the effect between the ventricle through the interventricular septum of reciprocal mechanical force transmission, allowing one ventricle to affect the other one’s functional performance. Therefore, the filing capacity of one ventricle is affected by the contractile force of the other one. The interventricular septum is the central part of this mechanism and each disease that affects the compliance and elasticity of the septum, as for HCM, decreases VI and, finally, RV function [5].

Our patient presented with RV dysfunction probably due to all these factors.

There are no specific treatment options for HCM patients with RV involvement. Generally, surgical treatments for HCM patients do not affect the progression of the disease [13] and may only lead to a relieving of symptoms and prevention of serious events.

RV involvement in HCM is associated with increased incidence of supraventricular and ventricular arrhythmias, severe dyspnea, pulmonary thromboembolism, progressive heart failure and increased risk of sudden cardiac death [4,14]; therefore, the RV should be routinely included in the detailed assessment of patients with HCM.

Since echocardiographic study of the right ventricle is challenging, the use of advanced imaging techniques is indicated.

A coronary artery fistula is an abnormal connection bypassing the myocardial capillary bed between a coronary and other cardiovascular structure including cardiac chamber (coronary–cameral fistulae) or blood vessels (arterio-venous fistulae) such as the coronary sinus, superior vena cava, or pulmonary artery [15]. The incidence is 0.1 to 0.2% in all patients undergoing coronary angiography. They most commonly affect the right coronary artery. The pathophysiological changes depend on the difference in pressure between the coronary artery and the site of drainage and on the resistance that blood meets along its course [16]. Considering the high RV pressure due to RVOT obstruction, this abnormality was firstly masked and underdetected, being visible only after pressure relief obtained with surgery.

According to the American Heart Association and American College of Cardiology guidelines for the management of adults with congenital heart disease [7], patients with small, asymptomatic fistulae should not be treated but managed with clinical follow-up, while small or medium-sized fistulae should be closed in case patients are symptomatic of myocardial ischemia, arrhythmias, ventricular dilatation or a dysfunction of uncertain origin. Our patient remained asymptomatic and we continued with sole follow-up over time.

The literature reports the presence of a coronary artery fistula in 0.3% of patients with congenital heart disease [16]. Its presence has been described in association with an apical form of HCM but it is unclear [17]. Its origin has been postulated to be due to the increased vascularization and fibrosis associated with hypertrophy or with congenital malformations leading to hypertrophy as a compensatory response to chronic volume overload caused by the shunt between the artery and ventricle [18].

Our case is interesting due to the association of two rare subtypes of a rare condition: coronary artery fistula and biventricular HCM with severe right ventricular obstruction.

## 4. Conclusions

Our patient underwent complete treatment and elimination of right ventricular obstruction, resulting in improved symptoms and a significant reduction in postoperative gradients. Direct relief of outflow tract obstruction can be achieved with low morbidity and good intermediate- to long-term results. Conventional surgery may provide significant symptomatic improvement and should thus be considered in the setting of HCM with outflow obstruction.

RV involvement in HCM has received little attention considering the prevalence of this disease. This may be due to previous technical limitations but, due to imaging improving and increased access in CMR, more data on RV in HCM have become available and need dedicated investigation. Clinicians need to be aware that possible RV involvement may coexist with LVH with special attention to its pathophysiological implications.

## Data Availability

Not applicable.
